# Early Safety Discrimination Under Uncertainty in Trait Anxiety: An Event-Related Potential Study

**DOI:** 10.3389/fnhum.2022.896211

**Published:** 2022-06-23

**Authors:** Yan Jin, Lei Zhang, Wei Chen, Xifu Zheng

**Affiliations:** ^1^School of Education Sciences, Huizhou University, Huizhou, China; ^2^Department of Psychology, South China Normal University, Guangzhou, China

**Keywords:** safety, uncertainty, high trait anxiety, event-related potentials, threat

## Abstract

Detection of safety-threat signals during uncertainty is an important mechanism of developmental anxiety disorder (AD). Although extensive research has focused on the detection of uncertain threat signals in anxious individuals, relatively little attention has been given to the identification of safety signals during uncertainty, which is an important way to relieve anxiety in individuals with AD. To investigate this phenomenon, 16 subjects with high trait anxiety (HTA) and 16 with low trait anxiety (LTA) completed a modified cue-target task in certain and uncertain stimulus blocks. In the uncertain block, the cue was followed by a threat picture or safety picture in 20% of trials, respectively; in the certain block, the cue could be followed by a threat picture or a safety picture on 100% of trials. Behavioral responses and event-related potentials (ERPs) were recorded. The ERP results demonstrated that LTA participants exhibited larger P2 amplitudes in the detection of safety cues than of threat cues during the uncertain block, whereas HTA participants showed significant P2 amplitudes between the safety and threat cues during the certain block, impairing the detection of safety stimuli during uncertainty. However, all participants exhibited greater N2 amplitudes following threat cues in certainty or uncertainty conditions. These findings pertaining to the P2 amplitude suggested distinctive attentional biases between HTA and LTA individuals, whereas the N2 amplitude showed association learning in uncertain conditions, compensating for safety-threat detection in HTA individuals.

## Introduction

According to environmental cues, safety-threat detection helps individuals to initiate adaptive behavioral responses. Previous studies have indicated that detection of threat stimuli during uncertainty, compared to certainty, increases behavioral avoidance and subjective distress (Shankman et al., 2011; Bennett et al., 2018). Cognitive neuroscience studies further show evidence that in anticipation of uncertain threat stimuli relative to certain threat stimuli, N100 and P3 (Nelson et al., 2015) were enhanced along with greater P2 (Huang et al., 2017) and SPN responses (Johnen and Harrison, 2020), eliciting larger insula and amygdala responses (Kastner-Dorn et al., 2018). These studies indicated that uncertainty alters threat anticipation processing.

Abnormal threat anticipation processing serves as the fundamental mechanism of anxiety disorders (ADs) (Geng et al., 2018). Some studies using subjective estimation have found that individuals with high trait anxiety (HTA) perceive future threat events as more likely to happen than do healthy controls (Castillo and Calvo, 2000). Neuroimaging studies provide further the evidence that individuals with HTA are associated with heightened anticipation activity of threat stimuli compared to that of safety stimuli (Veerapa et al., 2020). Compared to healthy controls, heightened reactivity to uncertain threat stimuli was more prominent in individuals with ADs (Simmons et al., 2013). Specifically, anxious individuals show increased amygdala activation (Williams et al., 2015), lower bed nucleus of the stria terminalis-amygdala connectivity (Clauss et al., 2019) in response to the uncertain threat cues, and positive correlation between state anxiety and N1 peak during the processing of uncertain cues (Yang et al., 2020).

These studies examined the behavioral and brain response to uncertainty cues, which were equally likely to be followed by threat or safety stimulus (Williams et al., 2015; Clauss et al., 2019), or only presented threat stimuli (Stegmann et al., 2019). Although the experimental paradigms were constructed using uncertain conditions, it was impossible to detect safety during the uncertain contexts. However, detecting safety signals under uncertainty is a ubiquitous way to relieve anxiety in daily life. For example, although novel coronavirus pneumonia may happen unpredictably, people may avoid misfortune by engaging in certain preventive behaviors, such as wearing masks and washing hands frequently. Further, a small number of studies have shown that experimental manipulation of safety learning reduces indices of state anxiety (Fonteyne et al., 2009; Cho, 2021). However, despite the importance of detection safety signals in uncertain situations, neural mechanisms to detect safety signals during uncertainty remain unclear.

Moreover, previous studies have found inconsistent results regarding anxious individuals utilizing safety cues (Spiegler et al., 2019). It was reported that safety signals reduced fear-potentiated startle in HTA individuals relative to LTA individuals (Jovanovic et al., 2005), whereas another study failed to reduce the fear response (Grillon and Ameli, 2001). In addition, theoretical paradigms also differ: some researchers have proposed a bottom-up processing mechanism in anxiety, where discrimination between safety and threat information occurs at the early stages of processing (Mogg and Bradley, 1998; Bar-Haim et al., 2007). Other studies hypothesized that distinguishing between safety and threat cues occur during the later stages of processing (Mogg et al., 2000), in which anxious individuals have top-down processing in threat-safety detection (Eysenck et al., 2007). These conflicting findings may reflect methodological differences (Denefrio et al., 2019).

Importantly, previous studies indicate that the effect of uncertainty on safety-threat detection seems to occur only in the first second after detection (Lin et al., 2015). In this case, event-related potentials (ERPs) can be sufficient to capture the time course of the uncertainty effect precisely and accurately, including two distinct processes at the early and late stages. Several electroencephalogram (EEG) components have been used to determine whether uncertainty-linked attention was affected, including P2 and N2. The first was P2, which is a positive-energy reading related to selective attention in early sensory processes, which peaks from 150 to 275 ms after stimulus presentation (Johnen and Harrison, 2020). One study found that emotionally uncertain cues elicited larger P2 amplitudes than did certain ones (Huang et al., 2017). Another study observed no P2 amplitude differences between the certain and uncertain conditions (Johnen and Harrison, 2020). Another ERP component, N2 is a negative-energy reading occurring over the frontal midline regions 200–350 ms after stimulation, reflecting attention control (Basten et al., 2011). Previous studies have found that N2 amplitudes under uncertain conditions are larger than those under certain ones (Gole et al., 2011), while other studies have found that N2 amplitudes under certain conditions are larger than those under uncertain ones (Lin et al., 2014; Peng et al., 2020). Thus, it is not clear whether anxiety influences safety-threat detection in the time course of brain activity.

Therefore, the present study combined the ERP technique (Yang et al., 2020) with the modified cue-target paradigm to examine the effect of trait anxiety on the sensitivity of safety-threat signals during uncertainty. This study is an extension of a previous one with more nuanced in the investigation of the effects of uncertainty on safety-threat detection in HTA individuals, which has been identified as a severe risk factor for ADs. Uncertainty was elicited by cues signaling different association degrees about whether a forthcoming stimulus would be safety or threat. Thus, the present study chose four graphics as detection cues, corresponding to 100% association safety condition (certain safety condition), 100% association threat condition (certain threat condition), 20% association safety condition (uncertain safety condition), and 20% association threat condition (uncertain threat condition). According to the safety signal hypothesis (Seligman and Binik, 1997), uncertainty makes safety signals lose safety function, and previous results report that anxiety-linked attention bias occurs at the early stage (Williams et al., 1992), hence, we hypothesized that, during the early stage, attention patterns would differ between the HTA and LTA groups. Meanwhile, based on the reinforcement sensitivity theory (Corr, 2002) that association learning enhances sensory discrimination of threat and safety cues (Kass et al., 2013; Stegmann et al., 2021), we hypothesized that at the later stage, HTA and LTA individuals would exhibit similar attention bias in the uncertain conditions.

## Materials and Methods

### Participants

High trait anxiety and LTA individuals aged 18–23 years were selected from a pool of nearly 400 undergraduate students based on their scores on the State-Trait Anxiety Inventory (STAI) trait scale scores, a self-evaluated questionnaire (Spielberger, 2010). Individuals scoring in the top and bottom 10 percentiles of the sample’s distribution were invited to participate in the experiment. Participants within these percentiles were only excluded if they had a history of an affective disorder. After two subjects were excluded due to excessive EEG artifacts, 16 participants were allocated to the HTA group [nine females, STAI score, mean ± standard deviation (SD): 54.62 ± 4.27), and 16 to the LTA group (eight females, STAI score: 27.5 ± 3.86]. There was a significant difference in STAI scores between the groups, *t*(30) = 29.07, *p* < 0.001. The groups did not differ in age, *t(*30*)* < 1. The mean age of the entire cohort was 20.16 ± 1.90 years. The study was approved by the Research Ethics Review Board of South China Normal University, and all participants signed an informed consent form for participation in the experiment.

### Procedure and Stimulus Materials

The stimuli and procedures were the same as those in our previous study (Jin et al., 2013). Subjects viewed 40 neutral and 40 negative pictures selected from the International Affective Picture System (IAPS) (Lang et al., 2008). The selection was based on normative valence and arousal evaluation using a 9-point scale ranging from “1” (extremely unpleasant) to “9” (extremely pleasant) and “1” (low excitement) to “9” (high excitement), respectively, as reported in the study by Lang et al. (2008). Neutral and negative pictures were selected for safety and threat stimuli respectively. In accordance with the IAPS scoring, comparison using *t*-tests indicated significant differences between the safety and threat stimuli in valence [threat: 1.88 ± 0.34, safety: 4.98 ± 0.32; *t*(78) = −41.69, *p* < 0.001] and in arousal [threat: 6.32 ± 0.63, safety: 2.92 ± 0.61; *t*(78) = 24.58, *p* < 0.001]. Stimuli were presented in two blocks. In the certain block, a square cue was always followed by the presentation of a threat picture or a hexagon cue was always followed by a safety picture. In the uncertain block, a circle cue was paired with a threat picture or a triangle cue was paired with a safety picture 20% of the time. A blank-screen appeared during the remaining 80% of time. There were four fixed associations between cues and pictures. The stimuli presentation within each block was randomized. There were 100 trials in both certain and uncertain blocks. The cues were randomly counterbalanced across subjects.

**FIGURE 1:**
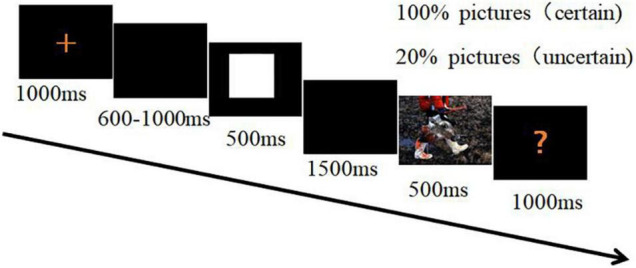
The procedure of the modified cue-target task.

A 500-ms cue signaled a safety or threat picture. The cue was followed by a 1,500-ms blank-screen interval before a threat or safety picture was shown. Each picture was shown for 500 ms and was followed by a red question mark on the screen. Termination of the red question mark was initiated by pressing a key within 1,000 ms. The subjects were required to indicate for each picture whether they perceived it as threat or safety (Figure 1). Half of the subjects were instructed to press the “F” key on the keyboard with their left index finger as accurately and quickly as possible following a threat picture, and to press the “J” key with their right index fingers when the red question mark followed a safety picture. The assignment of the response hands was reversed for the other half of the subjects. After each question mark, an inter-trial interval was randomly set for 4, 5, or 6 s.

### Electroencephalogram Recording

The EEGs were recorded with Brain Amp DC amplifiers. Recordings were made from 30 scalp locations in accordance with the international extended 10–20 system (FP1, FP2, F7, F3, Fz, F4, F8, FT7, FC3, FCz, FC4, FT8, T7, C3, Cz, C4, T8, TP7, CP3, CPz, CP4, TP8, P7, P3, Pz, P4, P8, O1, Oz, and O2). The left and right mastoids were recorded online, with the reference electrode attached over the left mastoid. The EEG data were re-referenced offline and calibrated to an averaged mastoid reference. The vertical electrooculogram (EOG) recording electrodes were positioned above and below the left eye, while the horizontal EOG recording electrodes were positioned at the outer canthus of both eyes. Scalp impedances were maintained below 5 kΩ. The signal was filtered offline using a band-pass of 0.1–30.0 Hz. The sampling rate was 500 Hz/channel. Trials with EOG artifacts (mean EOG voltage exceeding ± 75 μV) and those contaminated with artifacts due to peak-to-peak deflection exceeding ± 75 μV were excluded from averaging.

### Event-Related Potential Analyses

**FIGURE 2:**
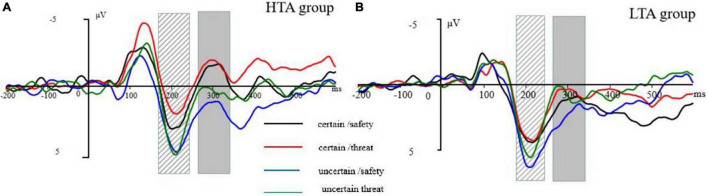
**(A)** Grand-averaged P2 waveform at electrode Cz. **(B)** Grand-averaged N2 waveform at electrode Cz. The striped column is P2 at 180–240 ms following the cue onset. The gray column is N2 at 270–340 ms following the cue onset. The cue-ERP for certain-safety (black line), certain-threat (red line), uncertain-safety (blue line), and uncertain-threat (green line) conditions in the high trait anxiety (HTA) group (left) and the low trait anxiety (LTA) group (right).

We only analyzed a cue-locked period because the identification of ERPs requires an adequate number of trials, which were not in the uncertain picture block. ERPs were time-locked to the cue onset. EEG activity was separately averaged for each condition (i.e., certain/threat, certain/safety, uncertain/threat, uncertain/safety). We used a 500-ms cue onset to evoke a priming effect. Based on Williams’ attentional bias of anxiety in the early aspects of processing, we did not expect to observe a slow wave (i.e., SPN) between the cue and target stimulus. Thus, the ERP epoch length was 700 ms with a pre-stimulus baseline of 200 ms. We observed the prominent N1 (time window: 110–150 ms), P2 (time window: 180–240 ms), and N2 (time window: 270–340 ms) components (see Figure 2). These components were robustly found in the midline, therefore, only three electrode sites (Fz, Cz, and Pz) were selected for analysis. A mixed-model analysis of variance (ANOVA) was used with the groups (two levels: HTA and LTA) as a between-subject factor, certainty (two levels: certain and uncertain), stimulus type (two levels: threat and safety), and electrode sites (three levels: Fz, Cz, and Pz) for ERP mean amplitudes as within-subject factors. As this study was interested in the effect of trait anxiety on the sensitivity of safety-threat signals during uncertainty, our analyses mainly focused on group-related interaction effects. The Greenhouse-Geisser correction was applied to adjust the degrees of freedom of the F-ratios.

## Results

### Behavioral Data

All subjects achieved a mean accuracy of 96.1% in identifying threat and safety stimuli, irrespective of certainty (certain or uncertain). Reaction time analysis was conducted on the trials with correct responses. The reaction time (mean ± standard deviation) of the HTA and LTA groups was: 305.03 ± 24.23 ms and 319.38 ± 24.17 ms, respectively. The difference between the groups was not significant.

### Event-Related Potential Data

In the N1 time window (110–150 ms), a mixed-model ANOVA conducted on the amplitudes demonstrated very significant main effects for type, *F*_(1_, _30)_ = 16.70, *p* < 0.001, η_*p*_^2^ = 0.36, with larger amplitudes for the threat (−2.00 ± 0.49 μV) than safety (−1.23 ± 0.49 μV) cues; electrode site, *F*_(2_, _60)_ = 10.45, *p* < 0.001, η_*p*_^2^ = 0.26, with Fz showing more negative amplitudes (−2.34 ± 0.60 μV) than Cz (−2.066 ± 0.54 μV) and Pz (−0.45 ± 0.49 μV). Furthermore, there was a significant electrode site by group interaction, *F*_(2_, _60)_ = 4.86, *p* < 0.05, η_*p*_^2^ = 0.14. The simple effect analysis of the electrode site by group interaction effect revealed no significant group effect. Although there was a significant three-way interaction of certainty by type by electrode site, *F*_(2_, _60)_ = 6.38, *p* < 0.05, η_*p*_^2^ = 0.18, no group interaction was observed for the component (Figure 2), therefore, N1 was not further addressed.

**FIGURE 3:**
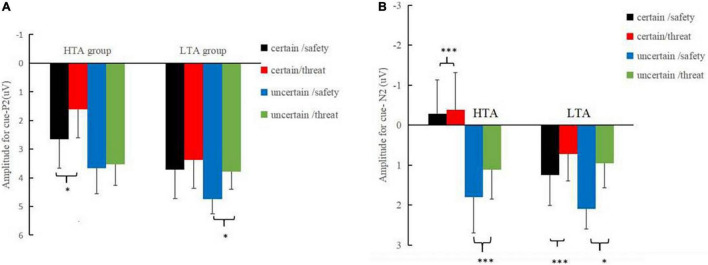
**(A)** Corresponding mean amplitudes and standard errors for cue-P2. **(B)** Corresponding mean amplitudes and standard errors for cue-N2. Certain-safety (black), certain-threat (red), uncertain-safety (blue), and uncertain-threat (green) trials, respectively in the LTA and HTA groups (*p < 0.05, ^***^p < 0.001).

Three main effects reached a significance level in the P2 time window (180–240 ms): type, *F*_(1_, _30)_ = 9.15, *p* < 0.01, η_*p*_^2^ = 0.23, with larger amplitudes for the safety (3.70 ± 0.46 μV) than threat (3.08 ± 0.45 μV) cues; certainty, *F*_(1_, _30)_ = 13.18, *p* < 0.001, η_*p*_^2^ = 0.31, with larger amplitudes for the uncertain condition (3.93 ± 0.48 μV) than the certain condition (2.84 ± 0.45 μV); and electrode site, *F*_(2_, _60)_ = 3.42, *p* < 0.05, η_*p*_^2^ = 0.10. Significant three-way interactions of certainty by electrode site by group [*F*_(2_, _60)_ = 3.43, *p* < 0.05, η_*p*_^2^ = 0.10] and type by certainty by group were observed [*F*_(1_, _30)_ = 4.48, *p* < 0.05, η_*p*_^2^ = 0.13] were significant. A further simple effect analysis (Figure 3A) indicated that, in the HTA group, the P2 amplitudes were larger for safety cues (2.66 ± 0.67 μV) than those for threat cues (1.61 ± 0.69 μV) under the certain condition, [*F*_(1_, _15)_ = 6.38, *p* < 0.05, η_*p*_^2^ = 0.40], while the amplitudes associated with safety (3.66 ± 0.90 μV) and threat (3.53 ± 0.74 μV) cues were comparable under the uncertain condition [*F*_(1_, _15)_ = 0.71, *p* > 0.05]. Analyses in the LTA group revealed a significant effect for the type under the uncertain condition, [*F*_(1_, _15)_ = 7.27, *p* < 0.05, η_*p*_^2^ = 0.25], indicating larger P2 amplitudes for safety cues (4.75 ± 0.51 μV) than threat cues (3.79 ± 0.61 μV).

The main effect of the threat cue (0.16 ± 0.65 μV) was significantly more negative than that of the safety cues (0.91 ± 0.64 μV) in the N2 time window [270–340 ms; *F*_(1_, _30)_ = 9.60, *p* < 0.01, η_*p*_^2^ = 0.24]. The certainty main effect indicated that the N2 amplitudes for an uncertain cue (1.27 ± 0.70 μV) were less negative than those of the certain cue [-0.20 ± 0.63 μV; *F*_(1_, _30)_ = 11.92, *p* < 0.01, η_*p*_^2^ = 0.28]. The site main effect differed significantly between the electrodes [*F*_(2_, _60)_ = 31.01, *p* < 0.001, η_*p*_^2^ = 0.51], with Fz showing more negative amplitudes (−1.62 ± 0.84 μV) than Cz (0.49 ± 0.75 μV) and Pz (2.74 ± 0.50 μV). More importantly, a four-way interaction of group, certainty, type, and electrode site was significant [*F*_(2_, _60)_ = 5.47, *p* < 0.01, η_*p*_^2^ = 0.15]. Further simple analysis (Figure 3B) found that the type effect was significant at Cz across certainty conditions: in the HTA group, a significantly larger N2 amplitude was observed in anticipation of a negative stimulus (−0.63 ± 1.12 μV) than for neutral ones (−0.49 ± 1.01 μV) during both the certain condition [*F*_(1_,_30)_ = 12.24, *p* < 0.001, η_*p*_^2^ = 0.01] and the uncertain condition [*F*_(1_,_30)_ = 14.31, *p* < 0.001, η_*p*_^2^ = 0.12], with a more negative N2 amplitude in anticipation of a threat stimulus (0.92 ± 1.21 μV) than for a safety one (1.65 ± 1.23 μV). Similarly, in the LTA group, a more negative N2 amplitude was observed in anticipation of a threat stimulus than a safety stimulus during the certain condition [*F*_(1_,_30)_ = 20.37, *p* < 0.001, η_*p*_^2^ = 0.07] and during the uncertain condition [*F*_(1_,_30)_ = 5.26, *p* < 0.05, η_*p*_^2^ = 0.45].

**FIGURE 4:**
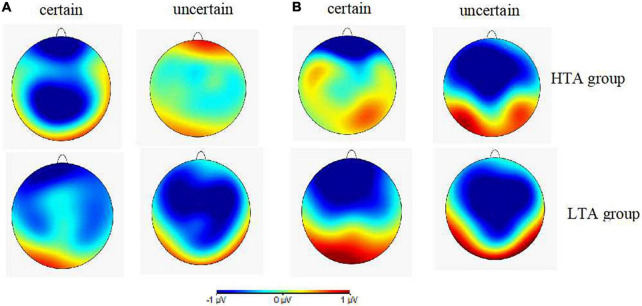
**(A)** Topographic maps showing voltage differences between certain and uncertain trials (threat minus safety) for cue-P2 at 180–240 ms after the cue onset. **(B)** Topographic maps showing voltage differences between certain and uncertain trials (threat minus safety) for cue-N2 at 270–340 ms after the cue onset for the HTA (up) and LTA (below) groups.

Figures 4A,B illustrates the topographies of difference waves (subtraction of the safety cue ERPs from the threat cue ERPs) at the 180–240 and 270–340 ms intervals after the cue onset. The various wave topographies at the 180–240 ms interval under the uncertain condition in the HTA group and under the certain condition in the LTA group were sky blue, indicating that the amplitudes generated by the threat and safety cues in this time window were similar, resulting in a difference of almost zero. ANOVA results for the wave difference at the 180–240 ms interval showed that the interaction between certainty, electrode site, and group was significant [*F*_(2_, _60)_ = 3.25, *p* < 0.05, η_*p*_^2^ = 0.10]. Simple effect analysis in the HTA group revealed that the amplitude at Pz was smaller under uncertain cues (−0.11 ± 0.41 μV) than certain ones (−1.67 ± 0.45 μV), *F*_(1,30)_ = 6.09, *p* < 0.05. This result was consistent with the P2 ERP results detailed above. ANOVA results for the wave differences at the 270–340 ms interval demonstrated a significant main effect for certainty [*F*_(1,30)_ = 15.66, *p* < 0.001, η_*p*_^2^ = 0.34]. The *post hoc* pairwise comparisons showed that the uncertain cues (−1.19 ± 0.24 μV) elicited larger N2 amplitudes than the certain ones (−0.31 ± 0.29 μV). Thus, topography of difference waves reflected distinct anticipation between the HTA an LTA groups during the different certainty contexts.

## Discussion

Using ERP measures, the present study investigated the effects of trait anxiety on safety-threat detection during an uncertain condition. P2 results revealed that individuals with LTA exhibited a higher anticipation for safety than for threat cues during the uncertain conditions, whereas HTA individuals showed significance between safety cues and threat cues during the certain conditions, and uncertain conditions elicited similar anticipation responses for the upcoming threat and safety stimuli, suggesting attention bias at the early stage. Moreover, N2 results showed that HTA individual discrimination between safety and threat cues was similar to those of LTA individuals, indicating that association learning enhances attention control during uncertainty in those with HTA.

The P2 is related to the allocation of attention in the early sensory processes (Gupta et al., 2019). Our study found a larger P2 amplitude during the uncertain condition than during the certain condition, which was consistent with the results of previous studies (Dieterich et al., 2016). Importantly, we found some evidence that HTA and LTA individuals have different attention allocation patterns during uncertainty. During uncertain conditions, LTA individuals showed differential threat-safety cue responses in P2 amplitudes. By contrast, HTA individuals showed no differential effects during uncertain conditions. These data suggest that LTA individuals demonstrate selective attention, which involves paying more attention to safety cues to ensure that these signals receive processing priority (Richards et al., 2014). Meanwhile, HTA individuals exhibited significant difference in P2 amplitudes during the certain condition, whereas they showed attention deficiency, which involves impaired discrimination between threat and safety cues during uncertain conditions; this was seen as no significant difference in P2 amplitudes. Our results were in line with those of a study that has shown that anxiety-liked individuals display increased threat generalization and less differentiation in uncertain contexts (Morriss et al., 2016). A similar effect has been observed for individuals with AD (Lissek et al., 2009; Grasser and Jovanovic, 2021). The safety-signal theory accounts for this observation (Seligman and Binik, 1997), which indicates that safety cues lose their safe signal function during uncertainty in individuals with HTA.

Interestingly, the uncertain cue-target picture pairing had a larger N2 amplitude for threat cues than for safety cues in both HTA and LTA participants. On one hand, this was a surprising finding, considering that N2 indicates inefficient attention control correlates with HTA (Bishop, 2009; Berggren and Derakshan, 2013). Uncertainty amplified the effects (Ran et al., 2016). The fact that both HTA and LTA individuals demonstrated high N2 amplitudes indicated significant discrimination between safety and threat cues. This may be because association learning strengthened affective electromyographic reactions in individuals with high anxiety (Corr, 2002), HTA individuals exhibited increased activations in the frontoparietal systems and had higher activation of the dorsolateral prefrontal cortex (Basten et al., 2011), which may be a compensatory mechanism for HTA individuals to achieve the same level of performance processing like LTA individuals.

In addition, although our ERP results revealed no group differences, HTA and LTA individuals demonstrated different initial safety sensitivity in P2, whereas in the N2 amplitudes, HTA and LTA individuals exhibited similar negative biases. A cognitive motivation of the anxiety model (Mogg and Bradley, 1998) is ideal to explain the differences and similarities in ERPs between the HTA and LTA individuals observed in this study. From the perspective of safety motivation, the safety-threat detection comprises the pre-evaluation stage, wherein subjects need to evaluate the stimulus based on the environmental cues. Previous studies have found that HTA individuals generated greater state anxiety than LTA individuals in an uncertain context (Williams et al., 2015), and uncertainty raises initial allocation attention bias (Peng et al., 2012), HTA individuals generalize anxiety, which disable the evaluation of the unpredictable stimuli, whereas LTA individuals use maximum attention resources to detect safety cues during uncertainty. However, after several trials, as all individuals build an association between the cue and target, they orient toward actual threat and direct their attention for the current goal task.

Some limitations of the present study should be mentioned. First, we studied the EEG response of HTA individuals and not subjects with clinically diagnosed ADs. This means that our current findings may not extend to individuals with ADs. Future studies should add such subjects to compare brain pattern differences among HTA individuals, LTA individuals and individuals with clinical anxiety to better explain this processing bias phenomenon. Second, our sample size was small. Future research should recruit higher numbers of HTA and LTA individuals. Third, although some studies (Bacigalupo and Luck, 2018) have shown that there was no significant difference in early and late trials of the association learning, EEG was averaged by early and late trials due to no time segments for the trials. Future studies will split into several sub-blocks to examine association learning dynamics of EEG effects.

P2 results revealed distinctive attentional biases between HTA and LTA individuals, whereas individuals with HTA exhibited negative biases in N2 like LTA individuals, in whom association learning may be a compensatory safety-threat detection mechanism.

## Data Availability Statement

The original contributions presented in this study are included in the article/supplementary material, further inquiries can be directed to the corresponding author.

## Ethics Statement

The studies involving human participants were reviewed and approved by the Research Ethics Review Board of South China Normal University (Approval Number: SCNU-PSY-2021-112). The patients/participants provided their written informed consent to participate in this study.

## Author Contributions

YJ and XZ: conceptualization, methodology, writing—original draft preparation and review and editing. LZ and WC: data collection. YJ: data analysis. XZ: supervision. All authors have read and agreed to the published version of the manuscript.

## Conflict of Interest

The authors declare that the research was conducted in the absence of any commercial or financial relationships that could be construed as a potential conflict of interest.

## Publisher’s Note

All claims expressed in this article are solely those of the authors and do not necessarily represent those of their affiliated organizations, or those of the publisher, the editors and the reviewers. Any product that may be evaluated in this article, or claim that may be made by its manufacturer, is not guaranteed or endorsed by the publisher.
